# Supplementary material for: Linear and inverted U-shaped dose-response functions describe estrogen effects on hippocampal activity in young women
Source: Nat Commun. 2018 Mar 23;9:1220. doi: 10.1038/s41467-018-03679-x (PMC5865215; doi:10.1038/s41467-018-03679-x)
Supplement: Supplementary file 1 — Supplementary Information(PDF 1157 kb) [file 41467_2018_3679_MOESM1_ESM.pdf]

Supplementary Information

Linear and inverted U-shaped dose-response functions describe estrogen effects on hippocampal activity in young women

Bayer et al

Supplementary Figures

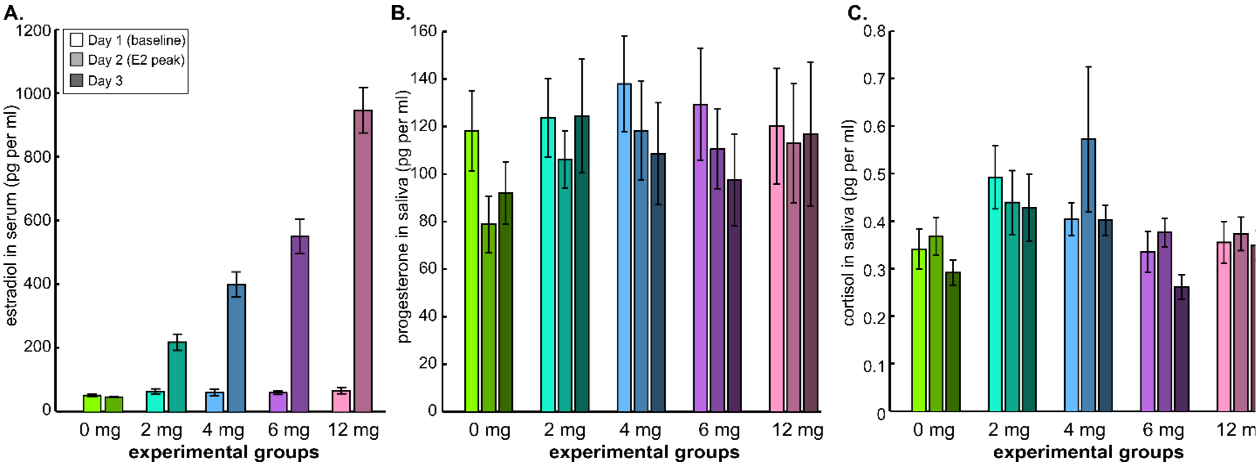

Supplementary Figure 1. Hormone levels in serum and saliva. Colors represent experimental groups (*N*=125) and color brightness represents testing days. Error bars represent standard errors of the mean. *A*. Concentration of 17-beta-estradiol (E2) in serum for experimental groups on Day 1 and Day 2 (no blood samples were collected on Day 3). Confirming the effectiveness of the pharmacological manipulation, the increases in salivary E2 levels from baseline (Day 1) to expected peak (Day 2) differed as intended between experimental groups [$p<.001$]. *B*. Concentration of progesterone in saliva for experimental groups. Groups did not significantly differ in Day1 to Day 2 changes in progesterone levels [$F(4,120)=.97$, $p=.426$]. Progesterone levels were considerably lower than during mid-luteal peak[1]. *C*. Concentration of cortisol in saliva for experimental groups. Groups did not significantly differ in Day 1 to Day 2 changes in cortisol levels [$F(4,120)=.94$, $p=.441$].

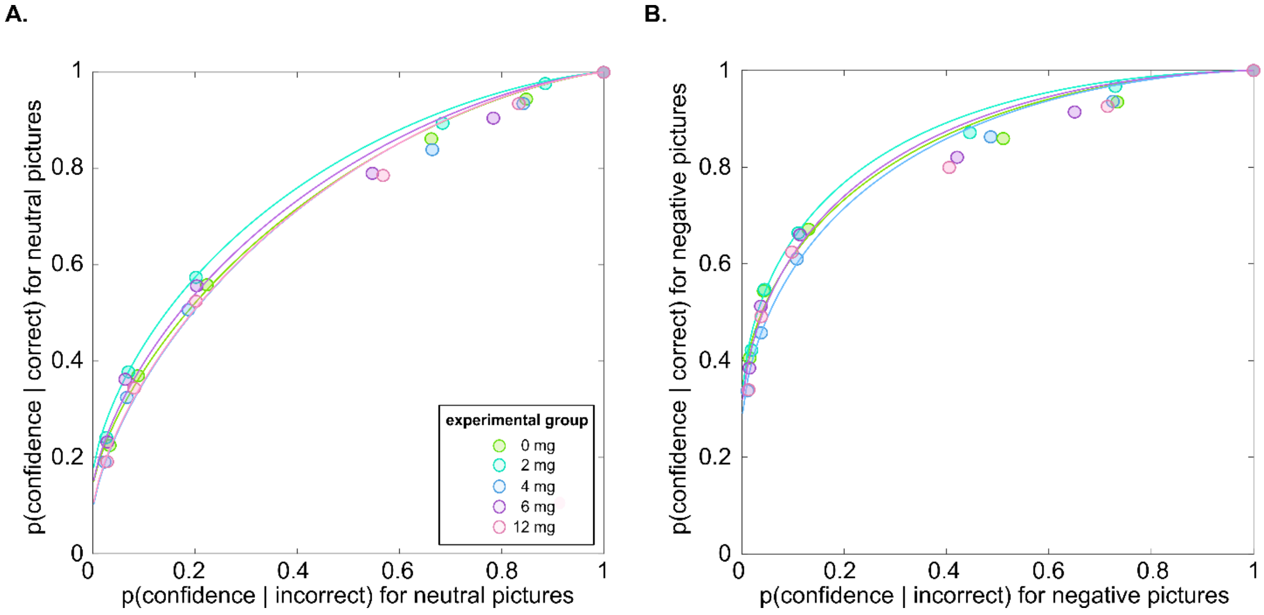

Supplementary Figure 2. Receiver-operating characteristic curves (ROC; *N*=123). ROC's are based on confidence ratings in the recognition memory task for neutral (A.) and negative pictures (B.).

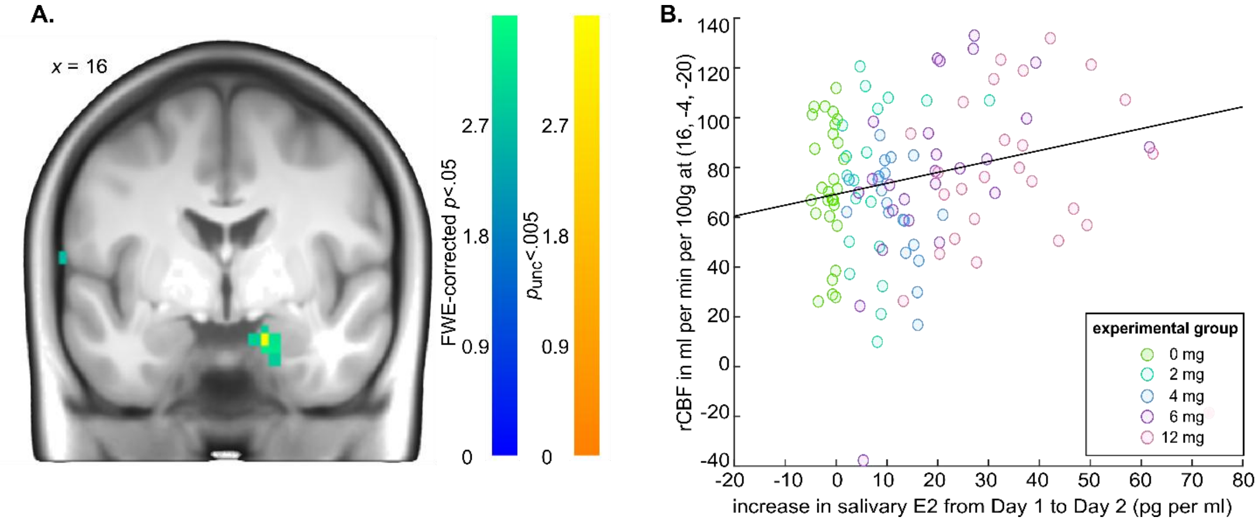

Supplementary Figure 3. Arterial spin labeling results. A. Statistical *t*-map of the positive linear relationship between salivary Day1 to Day2 increase in 17-beta-estradiol (E2) and regional cerebral blood flow (rCBF) in the right amygdala assessed by arterial spin labeling (*N*=117). This relationship did not survive family-wise error (FWE) correction for multiple comparisons [FWE-corrected *p*=.158; *t*-test]. The statistical map is thresholded at FWE-corrected *p*<.05 (warm color scale) and p_{unc}<.005 (cold color scale) for visualization purposes. B. Robust regression analysis between rCBF in ml per min per 100g extracted from the amygdala peak voxel (*y*-axis) and salivary E2 increases (*x*-axis) confirmed the positive linear relationship. Colors represent experimental groups.

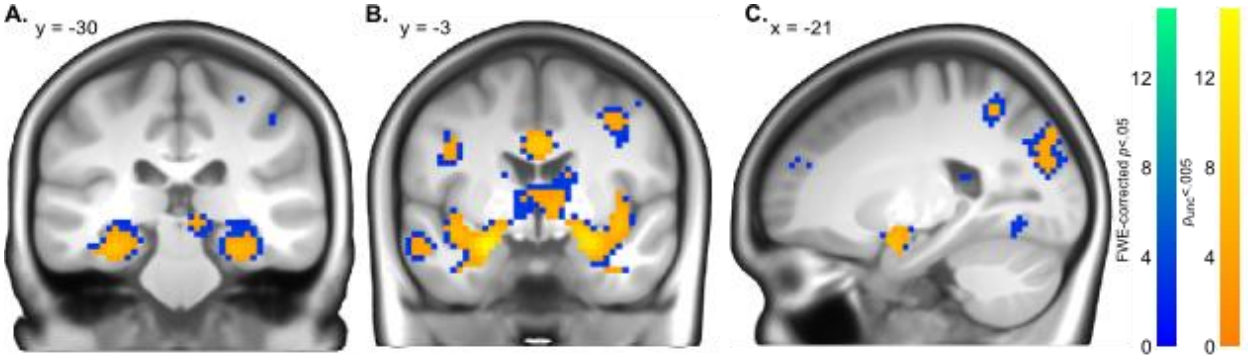

Supplementary Figure 4. Statistical *t*-maps for main effects of the emotional memory paradigm. Statistical maps are thresholded at FWE-corrected $p<.05$ (warm color scale) and $p_{unc}<.005$ (cold color scale) for visualization purposes. *A.*The remember>know contrast (*N*=118) showed robust effects in the left and right hippocampus [FWE-corrected $p<.001$; *t*-test]. *B.* The negative>neutral contrast yielded robust main effects in the bilateral amygdala [*N*=121; FWE-corrected $p<.001$; *t*-test]. *C.* The emotional enhancement of memory (EEM; hit > miss x negative > neutral) contrast (*N*=121) showed main effects were observed bilaterally in the hippocampus [right: FWE-corrected $p=.009$, left: FWE-corrected $p=.021$; *t*-test] and the amygdala [FWE-corrected $p<.001$; *t*-test].

Supplementary Tables

Supplementary Table 1. Descriptive and inferential statistics of sample characteristics

	experimental group					statistical analyses	
	**0 mg**	**2 mg**	**4 mg**	**6 mg**	**12 mg**	***F***	***p***
age (years)	25.57 ± 3.41	26.38 ± 3.85	26.50 ± 4.03	25.48 ± 4.15	25.83 ± 3.71	.34	.848
weight (kg)	61.47 ± 6.58	62.90 ± 8.62	64.85 ± 10.72	60.76 ± 8.82	66.17 ± 10.34	1.69	.157
BMI	21.83 ± 2.33	22.17 ± 2.14	23.48 ± 3.68	21.75 ± 2.93	22.71 ± 3.35	1.08	.372
						X²	***p***
education (n cases)						14.6	.558
~10 y	0	0	2	0	1		
~11 y	1	0	0	0	0		
~12 y	2	1	0	1	1		
~13-16.5 y	18	13	8	16	15		
> 16.5 y	9	7	10	8	12		
nulliparity	28	19	19	23	28	.94	.919

All analyses are based on *N*=125. BMI=body-mass-index. Women were considered as ‘nulliparous’ when they have never been pregnant for longer than 8 weeks. Welch’s Robust Test for Equality of Means was calculated for the variable BMI because of inhomogeneous variances across groups (see Supplementary Note 1).

Supplementary Table 2. Interquartile ranges (maximum-minimum) and Cohen's *d* for increases in salivary 17-beta-estradiol (E2) from Day 1 to Day 2 (*N*=125)

	**Day1 to Day2 change in salivary E2**	
**experimental group**	**interquartile ranges**	***d***
0 mg	2.23	-0.48
2 mg	6.31	1.48
4 mg	6.58	2.67
6 mg	17.25	2.34
12 mg	16.33	3.14

Supplementary Table 3. Principal Component Analysis of mood ratings.

	loadings > |.5|				
**dimension**	**component 1**	**component 2**	**component 3**	**component 4**	**communality**
alert/drowsy	.782				.675
strong/feeble	.777				.702
energetic/lethargic	.767				.660
clear-headed/muzzy	.744				.672
quick witted/mentally slow		.805			.746
attentive/dreamy		.734			.634
interested/bored		.700			.605
gregarious/withdrawn		.625			.559
proficient/incompetent		.558			.456
relaxed/tensed			.799		.658
tranquil/troubled			.792		.711
calm/excited			.722		.566
anxious/not anxious			-.655		.441
contended/discontended			-.510		.508
well-coordinated/clumsy				.683	.644
amicable/antagonistic				.626	.600
happy/sad		.506		.572	.615
**eigenvalue**	3.015	2.954	2.805	1.678	
**% of total variance**	17.737	17.375	16.498	9.870	
**total variance**				**61.480**	

The dimension happy/sad was assigned to component 4 based on semantic coherence.

Supplementary Table 4. Descriptive and inferential statistics for recognition performance in the emotional memory task (N=123)

	**Means and Standard Deviations**					**Robust Regression**					
	**experimental group**					**linear**			**quadratic**		
	**0 mg**	**2 mg**	**4 mg**	**6 mg**	**12 mg**	t	p_{corr}	β_{std}	t	p_{corr}	β_{std}
neutral pictures											
hit rate	.56 ± .16	.57 ± .18	.51 ± .19	.56 ± .17	.52 ± .16	-1.08	.562	-.102	-.15	>.999	-.036
false alarm rate	.22 ± .12	.20 ± .12	.19 ± .08	.20 ± .12	.20 ± .09	-.56	>.999	-.047	-.33	>.999	-.071
recollection	.14 ± .14	.17 ± .10	.09 ± .10	.14 ± .12	.10 ± .10	-1.50	.272	-.130	-.18	>.999	-.040
familiarity	.69 ± .37	.81 ± .28	.73 ± .41	.75 ± .36	.73 ± .30	.03	>.999	.003	.33	>.999	.081
dprime	.99 ± .38	1.11 ± .34	.94 ± .42	1.06 ± .43	.95 ± .37	-.36	>.999	-.034	.21	>.999	.053
criterion	.33 ± .39	.36 ± .45	.46 ± .37	.37 ± .39	.41 ± .36	1.07	.578	.095	.37	>.999	.085
area under the curve	.70 ± .08	.72 ± .08	.69 ± .10	.71 ± .08	.70 ± .07	-.43	>.999	-.042	.31	>.999	.077
meta dprime	.76 ± .41	.77 ± .20	.70 ± .48	.74 ± .41	.69 ± .34	-1.11	.536	-.099	-.05	>.999	-.012
meta criterion	.32 ± .39	.35 ± .44	.45 ± .36	.36 ± .38	.41 ± .35	1.06	.580	.095	.36	>.999	.084
negative pictures											
hit rate	.67 ± .16	.66 ± .18	.61 ± .18	.66 ± .13	.62 ± .16	-1.39	.336	-.131	-.50	>.999	-.121
false alarm rate	.13 ± .10	.11 ± .11	.11 ± .08	.11 ± .08	.10 ± .07	-.61	>.999	-.125	-.61	>.999	-.125
recollection	.33 ± .19	.32 ± .20	.27 ± .17	.30 ± .17	.29 ± .14	-.92	.722	-.087	-.10	>.999	-.025
familiarity	1.10 ± .41	1.25 ± .51	1.12 ± .42	1.17 ± .44	1.09 ± .42	-.68	>.999	-.065	-.23	>.999	-.058
dprime	1.74 ± .49	1.87 ± .56	1.67 ± .50	1.76 ± .57	1.80 ± .62	-.63	>.999	-.059	.51	>.999	.121
criterion	.38 ± .42	.46 ± .46	.53 ± .42	.44 ± .31	.56 ± .39	1.36	.350	.124	.60	>.999	.142
area under the curve	.82 ± .08	.83 ± .08	.80 ± .10	.82 ± .07	.80 ± .09	-.87	.770	-.082	-.16	>.999	-.038
meta dprime	1.12 ± .62	1.16 ± .52	1.17 ± .49	1.17 ± .60	.99 ± .45	-1.86	.130	-.159	-.33	>.999	-.073
meta criterion	.37 ± .41	.44 ± .45	.51 ± .40	.43 ± .30	.54 ± .38	1.34	.368	.121	.58	>.999	.136

The process parameters recollection and familiarity as well as the area under the curve, meta dprime and the meta criterion were estimated using the confidence ratings. All analyses were Bonferroni corrected for multiple comparisons (i.e. the two valence categories; p_{corr}). Meta dprime and the meta criterion were calculated using Matlab scripts published by Fleming and colleagues[2].

Supplementary Table 5. Median number and range of hit and correct rejection trials (*N*=123)

	**experimental group**									
	**0 mg**		**2 mg**		**4 mg**		**6 mg**		**12 mg**	
	**median**	**range**	**median**	**range**	**median**	**range**	**median**	**range**	**median**	**range**
**neutral pictures**										
hits	51.00	66.00	55.00	58.00	42.00	54.00	51.00	59.00	50.00	58.01
correct rejections	70.50	45.00	75.00	39.01	75.00	25.99	73.00	50.00	72.00	34.00
**negative pictures**										
hits	58.50	56.00	66.00	55.00	60.50	56.01	59.00	41.00	60.00	52.99
correct rejections	80.00	37.00	84.00	44.00	81.50	32.00	81.00	29.00	82.00	22.00

The term ‘range’ refers to the difference between the highest and lowest value within each experimental group.

Supplementary Table 6. Descriptive and inferential statistics of confidence ratings in the emotional memory task

	Means and Standard Deviations					Robust Regression					
	experimental group					linear			quadratic		
	**0 mg**	**2 mg**	**4 mg**	**6 mg**	**12 mg**	t	p_{corr}	β_{std}	t	p_{corr}	β_{std}
**neutral pictures**											
confidence targets, 'old' responses	2.03 ± .42	2.02 ± .35	1.96 ± .34	2.03 ± .32	1.97 ± .33	-.61	>.999	-.057	.43	>.999	.105
confidence targets, 'new' responses	1.46 ± .41	1.36 ± .26	1.42 ± .43	1.65 ± .47	1.55 ± .43	1.37	.346	.104	.72	.946	.143
confidence lures, 'old' responses	1.57 ± .36	1.45 ± .34	1.48 ± .27	1.43 ± .27	1.50 ± .35	-.65	>.999	-.051	-.85	.794	-.174
confidence lures, 'new' responses	1.63 ± .46	1.53 ± .30	1.58 ± .49	1.83 ± .50	1.74 ± .42	1.26	.420	.112	1.00	.640	.230
**negative pictures**											
confidence targets, 'old' responses	2.39 ± .31	2.42 ± .36	2.25 ± .39	2.33 ± .29	2.28 ± .29	-1.79	.150	-.160	-.41	>.999	-.094
confidence targets, 'new' responses	1.61 ± .48	1.55 ± .28	1.54 ± .50	1.74 ± .49	1.71 ± .41	1.66	>.999	.155	.63	>.999	.151
confidence lures, 'old' responses	1.51 ± .43	1.59 ± .49	1.35 ± .29	1.52 ± .39	1.52 ± .49	-.86	.782	-.072	-.06	>.999	-.014
confidence lures, 'new' responses	1.86 ± .49	1.91 ± .45	1.87 ± .56	2.04 ± .57	1.96 ± .45	.96	.674	.095	.59	>.999	.151

All analyses are based on N=123, except for analyses on confidence ratings for negative lure items judged as 'old' which is based on N=118 because of missing responses within this category (missing data: n=1 in 0 mg, n=1 in 2 mg, n=1 in 6 mg and n=2 in 12 mg group). Confidence ratings were coded as follows: ‘absolutely sure old/new’=3, ‘relatively sure old/new’=2, ‘unsure old/new’=1. All analyses were Bonferroni corrected for multiple comparisons (i.e. the two valence categories; p_{corr}).

Supplementary Table 7. Descriptive and inferential statistics of reaction times for the encoding task (inside/outside decision) in ms

	**Means and Standard Deviations**					**Robust Regression**					
	**experimental group**					**linear**			**quadratic**		
	**0 mg**	**2 mg**	**4 mg**	**6 mg**	**12 mg**	t	p_{corr}	β_{std}	t	p_{corr}	β_{std}
**neutral targets**											
remember	988.3 ± 153.1	983.4 ± 167.4	948.0 ± 190.5	942.1 ± 172.9	973.9 ± 118.5	-1.10	.550	-.089	.28	>.999	.058
know	982.0 ± 135.1	944.3 ± 95.8	919.1 ± 180.1	972.6 ± 190.7	1026.4 ± 139.6	-.16	>.999	-.014	-.35	>.999	-.078
new	965.3 ± 120.3	941.7 ± 116.9	911.1 ± 156.3	934.9 ± 174.9	1030.3 ± 195.1	-.58	>.999	-.049	.02	>.999	.005
**negative targets**											
remember	1172.3 ± 179.1	1104.4 ± 141.0	1062.1 ± 185.8	1110.7 ± 233.2	1113.0 ± 140.6	-1.91	.118	-.172	-1.34	.368	-.305
know	1106.9 ± 173.8	1116.2 ± 162.6	1017.8 ± 178.5	1081.7 ± 191.3	1135.0 ± 173.6	-.68	.994	-.066	.76	.894	.186
new	1113.9 ± 180.0	1069.0 ± 130.6	1010.0 ± 162.1	1096.8 ± 202.1	1137.7 ± 174.8	-.17	>.999	-.015	-.36	>.999	-.085

All analyses were Bonferroni corrected for multiple comparisons (i.e. the two valence categories; p_{corr})

Supplementary Table 8. Descriptive and inferential statistics of arousal ratings as a function of responses in the recognition memory task

	**Means and Standard Deviations**															**Robust Regression**					
	**experimental group**															**linear**			**quadratic**		
	**0 mg**			**2 mg**			**4 mg**			**6 mg**			**12 mg**			***t***	p_{corr}	β_{std}	***t***	p_{corr}	β_{std}
**neutral pictures**																					
total	2.49	±	1.11	2.28	±	1.05	3.04	±	1.21	3.20	±	1.29	3.28	±	1.05	2.72	.015	.257	1.51	.267	.366
remember responses	2.69	±	1.15	2.94	±	1.35	3.05	±	1.18	3.49	±	1.38	3.55	±	1.07	2.09	.077	.203	1.67	.196	.409
know responses	2.51	±	1.13	2.31	±	1.13	2.98	±	1.25	3.20	±	1.39	3.24	±	1.09	2.68	.017	.258	1.67	.196	.414
new responses	2.42	±	1.14	2.06	±	1.08	2.96	±	1.24	3.03	±	1.20	3.20	±	1.15	2.54	.025	.240	1.31	.386	.319
**negative pictures**																					
total	5.92	±	1.11	5.70	±	1.16	5.76	±	1.44	6.48	±	.91	6.26	±	.87	1.13	.524	.102	.54	>.999	.126
remember responses	6.49	±	1.11	6.48	±	1.10	6.32	±	1.50	6.98	±	.81	6.73	±	.81	.83	.818	.073	-.17	>.999	-.039
know responses	5.76	±	1.22	5.71	±	1.29	5.65	±	1.53	6.26	±	.99	6.08	±	.86	1.15	.508	.104	.71	.958	.166
new responses	5.19	±	1.35	5.00	±	1.36	5.32	±	1.57	5.97	±	1.19	5.84	±	1.05	1.67	.196	.157	1.06	.580	.257

All analyses are based on N=123, except for analyses on confidence ratings for negative target items judged as 'remember' which are based on N=118 because of missing responses within this category (missing data: n=1 from 0 mg, n=1 from 2 mg, n=1 from 6 mg and n=2 from 12 mg group). All analyses were Bonferroni-corrected for multiple comparisons (i.e. the two valence categories; p_{corr})

Supplementary Table 9. Brain regions associated to the remember>know contrast family-wise error corrected for the whole scan volume (*N*=118)

	main effect: remember>know					
**region**	***x***	***y***	***z***	***Z***	**FWE- corrected *p***	***k* at FWE- corrected p <.05**
**left hemisphere**						
medial temporal cortex	-30	-30	-18	7.39	<.001	419
precuneus	-6	-54	12	6.33	<.001	49
superior parietal cortex	-42	-75	18	5.82	<.001	105
inferior frontal operculum	-39	6	24	5.79	<.001	27
orbito-frontal gyrus	-39	30	-18	5.58	.001	34
medial frontal gyrus	-3	42	-18	5.39	.002	31
inferior frontal gyrus	-48	30	12	5.37	.002	18
**right hemisphere**						
inferior frontal gyrus	48	36	9	7.55	<.001	176
inferior temporal gyrus	48	-57	-12	7.73	<.001	109
superior parietal cortex	30	-78	30	6.85	<.001	241
medial temporal cortex	30	-42	-9	6.78	<.001	204
orbito-frontal gyrus	27	30	-15	6.21	<.001	18
medial temporal gyrus	54	-6	-18	5.69	<.001	13
calcarine sulcus	18	-51	9	5.42	.001	17
posterior cingulate	9	-54	12	4.85	.023	2

Supplementary Table 10. Brain regions associated to general encoding success (i.e. subsequent hits>misses) family-wise error corrected for the whole scan volume (*N*=121).

	main effect: hit>miss					
**region**	***x***	***y***	***z***	***Z***	**FWE- corrected *p***	***k* at FWE-corrected p <.05**
**left hemisphere**						
medial temporal lobe	-27	-39	-15	8.70	<.001	1270
middle & inferior frontal gyrus	-36	33	-15	8.40	<.001	358
superior frontal gyrus	-6	57	27	7.29	<.001	92
precuneus	-18	-54	9	6.40	<.001	70
middle temporal gyrus	-54	-9	-18	6.38	<.001	56
superior temporal gyrus	-45	18	-21	6.21	<.001	8
corpus callosum	-6	6	24	4.78	.028	1
**right hemisphere**						
middle & inferior frontal gyrus	42	12	24	9.21	<.001	541
medial temporal lobe	21	-9	-21	8.82	<.001	1280
medial frontal gyrus	3	42	-18	7.93	<.001	112
middle temporal gyrus	57	-3	-18	7.27	<.001	63
precuneus	15	-51	12	6.94	<.001	94
superior temporal gyrus	45	18	-33	6.59	<.001	16
thalamus	3	-18	6	5.63	<.001	15
middle frontal gyrus/precentral gyrus	48	-3	51	5.43	.001	14
inferior occipital	27	-93	-6	4.74	.032	2

Supplementary Table 11. Cluster extents and results of robust regression analyses using the increase in salivary Day 1 to Day 2 increase in 17-beta-estradiol (E2) and contrast estimates from the respective peak voxels.

	**SPM analyses**	**Robust Regression**			
**relationships with salivary Day 1 to Day 2 E2 increases**	**k at p_{unc}<.005**	t	p_{unc}	**Δ BIC**	β_{std}
**linear**					
remember > know contrast in the hippocampus (33, -30, -12)	57	3.57	<.001	-4.55	.315
negative > neutral contrast in the precuneus (6, -60, 15)	345	-4.18	<.001	-6.72	-.328
negative > neutral contrast in the brainstem (6, -30, -12)	118	-4.01	<.001	-5.79	-.311
PPI for negative > neutral contrast for precuneus-brainstem connectivity (6, -21, -9)	35	-3.01	.003	-2.85	-.256
**quadratic**					
remember > know contrast in the hippocampus (-15, -33, -12)	29	4.36	<.001	8.50	.834
EEM contrast in the insula (42, -6, 9)	433	2.49	.014	16.94	.505

ΔBIC = Difference in Bayesian Information Criterion (BIC) between linear and quadratic models. EEM = emotional enhancement of memory (hit > miss x negative > neutral). All analyses are based on N=121, except of analyses using the remember>know contrast which are based on N=118.

Supplementary Table 12. Cluster showing relationships to the Day 1 to Day 2 increase in salivary 17-beta-estradiol (E2) surviving a threshold of p_{unc} < .001 and cluster extent k $\geq$ 10 voxels.

**contrast/region**	***x***	***y***	***z***	***Z***	***k* at p_{unc} <.001**
**remember > know: negative linear relationship**					
calcarine sulcus	18	-75	12	3.97	27
**hit > miss: positive linear relationship**					
superior parietal gyrus	24	-63	36	3.89	30
supramarginal gyrus	45	-42	33	3.69	13
putamen	24	6	12	3.59	23
medial frontal gyrus	6	27	45	3.34	12
**hit > miss: negative linear relationship**					
insula	39	0	-15	4.43	49
posterior cingulate/calcarine sulcus	24	-57	3	4.14	80
posterior cingulate/calcarine sulcus	-15	-66	9	4.00	52
**negative > neutral: positive linear relationship**					
precentral gyrus	-42	-15	57	3.66	14
**negative > neutral: negative linear relationship**					
superior frontal gyrus	24	6	48	4.17	11
**negative > neutral: negative quadratic relationship**					
corpus callosum	0	-24	15	3.73	18
**EEM effect (hit > miss x negative > neutral): positive linear relationship**					
inferior frontal gyrus	57	9	18	4.10	20
middle occipital gyrus	-39	-87	3	3.72	22
fusiform gyrus	36	-81	-18	3.69	10
middle frontal gyrus	-24	-15	63	3.61	10
**EEM effect (hit > miss x negative > neutral): negative linear relationship**					
brainstem	6	-30	-12	4.41	72
superior temporal gyrus	-51	-42	12	3.98	19
precuneus/calcarine sulcus	-15	-69	15	3.90	89
thalamus	-12	-30	12	3.65	16
caudate	-12	3	6	3.60	11
medial frontal gyrus	-3	18	42	3.55	34
**EEM effect (hit > miss x negative > neutral): negative quadratic relationship**					
thalamus	12	-15	6	3.93	21
superior temporal gyrus	-63	-18	3	3.81	12
cerebellum	0	-45	-33	3.80	13

All analyses are based on *N*=121, except of analyses using the remember>know contrast which are based on *N*=118.

Supplementary Table 13. Number of cases included in each analysis.

	***n* per experimental group**					***N***
	**0**	**2**	**4**	**6**	**12**	**Σ**
full sample	30	21	20	25	29	125
behavioral analyses, hormone levels	30	21	20	25	27	123
**MRI**						
functional imaging: negative vs. neutral, hits vs. misses, EEM contrast	30	20	20	25	26	121
functional imaging: remember vs. know	29	20	20	24	25	118
arterial spin labeling	28	19	20	25	25	117

Supplementary Note 1

Levene's test for homogeneity of variances indicated inhomogeneous variances across groups for the body-mass-index (BMI) [$F(4,120)=3.267$, $p=.014$]. Welch's Robust Test of Equality of Means was therefore calculated to test for group differences in BMI (Supplementary Table 1). There was no evidence for inhomogeneous variances for the variables age [$F(4,120)= .537$, $p=.709$] or weight [$F(4,120)=1.94$, $p=.108$].

To explore whether individual differences in age might contribute to the variability in Day 1 to Day 2 E2 increase, we calculated a multiple robust regression analysis using mg per kg, age and the interaction term mg per kg*age as predictors for salivary Day 1 to Day 2 increase. However, neither age [$t(123)=-.04$, $p=.964$, $\beta_{std}=-.002$] nor mg per kg*age [$t(123)=-.68$, $p=.501$, $\beta_{std}=-.03$] reached significance.

Supplementary Note 2

Levene's test for homogeneity of variances indicated inhomogeneous variances across groups for Day 1 to Day 2 changes in salivary and serum E2 [saliva: $F(4,120)=10.66$, $p<.001$; serum: $F(4,120)=12.68$, $p<.001$]. There was no evidence for inhomogeneous variances for Day 1 to Day 2 changes for progesterone or cortisol [progesterone: $F(4,120)=1.66$, $p=.165$, cortisol: $F(4,120)=1.15$, $p=.339$].

Supplementary Note 3

One-sample *t*-tests against .5 confirmed that hit rates [neutral: $t(122)=2.94$, $p=.004$; negative: $t(122)=10.17$, $p<.001$] as well as correct rejection rates [neutral: $t(122)=48.36$, $p<.001$; negative: $t(122)=30.92$, $p<.001$] were significantly above chance levels.

Supplementary Note 4

In order to understand why higher E2 levels enhanced arousal for neutral pictures, we tested the following two hypotheses. First, E2 might increase the ‘emotional carry effect’, that is, arousal elicited by a negative picture could influence the rating of the next neutral picture. To test this possibility, individual arousal ratings for neutral stimuli following another neutral stimulus (‘neutral-after-neutral’) and neutral stimuli directly following a negative stimulus (‘neutral-after-negative’) were each averaged for every participant. All statistical analyses in this section were Bonferroni corrected for multiple comparisons (denoted by p_{corr}). Consistent with an emotional carry-over effect, neutral-after-negative stimuli [M=2.89, SD=1.19] were generally perceived as more arousing than neutral-after-neutral stimuli [M=2.79, SD=1.22; $t(122)$=3.76, p<.001]. However, robust regression analyses showed significant associations between salivary increase in E2 from Day 1 to Day 2 and arousal ratings for both neutral-after-negative [$t(121)$=2.75, p_{corr}=.014. β_{std}=.261] as well as neutral-after-neutral [$t(121)$=2.65, p_{corr}=.018, β_{std}=.251] pictures. Moreover, the emotional carry-over effect (arousal neutral-after-negative − arousal neutral-after-neutral) was not significantly associated to E2 [$t(121)$=.190, p_{corr}>.999, β_{std}=.017]. Thus, although carry-over effects were present in our data, they did not explain the effects of E2 on arousal ratings for neutral pictures.

Second, it is possible that viewing negative pictures might induce a negative mood over time, which in turn leads to greater arousal also with neutral pictures (‘mood induction effect’), an effect which might be increased by E2. Evidence that the mood induction effect could be enhanced by E2 is provided by Pruis and colleagues ([3]; 2nd experiment). The authors found that women under HRT (compared to those not undergoing HRT) rated the neutral beginning of a story as less arousing than the also neutral third section, which was preceded by a negative story section. Our additional analyses corroborated the mood-induction hypothesis: arousal ratings for

neutral pictures from the first third of the pictures seen were significantly lower than from the last third [$t(122)=-2.19$, $p=.030$]. Critically, the difference of arousal ratings for neutral pictures (last third – first third) was positively associated with salivary E2 Day 1 to Day 2 increase [$t(121)=3.09$, $p_{corr}=.005$, $\beta_{std}=.148$]. In other words, the higher the increase in salivary E2, the higher the increase in arousal ratings for neutral pictures over time. As such, it is quite plausible that the increase in E2 increased mood induction effects and thereby arousal ratings for neutral pictures.

Supplementary Note 5

To test whether the relationship between salivary Day 1 to Day 2 E2 increases and brain activity associated to the remember>know contrast is specific for hippocampal regions, we created masks from the superior parietal as well as the inferior frontal clusters associated to the main effect of the remember>know contrast (thresholded at FWE-corrected p>.05; t-test; N=118; see Supplementary Table 9). These regions have been chosen based on previous reports of hormonal effects in these areas [4–6]. However, whole-brain regression analyses did not show a significant relationships family-wise error corrected within the single respective masks. In detail, there was neither a significant linear or quadratic relationship in the right parietal [linear: (33, -81, 36), Z=1.49, FWE-corrected p=.872; quadratic: (24, -66, 48), Z=.68, FWE-corrected p=.976; t-tests; N=118], left parietal [linear: (-36, -87, 15), Z=2.40, FWE-corrected p=.220; quadratic: (-45, -78, 18), Z=2.39, FWE-corrected p=.209; t-tests; N=118], right inferior frontal [linear: (42, 6, 24), Z=1.33, FWE-corrected p=.845; quadratic: (54, 36, 3), Z=.72, FWE-corrected p=.836; t-tests; N=118] or left inferior frontal cluster [linear: (-48, 33, 12, Z=.30, FWE-corrected p=.630; quadratic: (-45, 33, 12), Z=-.22, FWE-corrected p=.678; t-tests; N=118]. As Ottowitz and colleagues [5] also report significant effects of E2 in two further prefrontal areas, we searched for relationships between salivary Day 1 to Day 2 increase and remember-know associated brain activity within a 5 mm sphered centered around the reported peaks. Again, no relationship turned out to be significant either within the right superior frontal gyrus [linear: (15, 60, 24), Z=1.20, FWE-corrected p=.405; quadratic: (8, 57, 24), Z=0.25, FWE-corrected p=.632; t-tests; N=118] or right middle frontal gyrus [(39, 18, 51), Z=-.20, FWE-corrected p=.679; quadratic: (36, 18, 51), Z=-.26, FWE-corrected p=.672; t-tests; N=118] small volume corrected within the respective sphere. As such, current data suggest that the linear and quadratic relationships between salivary

E2 increase and brain activity associated to the remember > know contrast are specific to hippocampal regions.

Supplementary Note 6

To explore hippocampal functional connectivity associated to the remember>know contrast, we created a mask from the hippocampal cluster showing a linear relationship to salivary E2 increase [(33, -30, -12) thresholded at $p_{unc}<.001$; *t*-test; *N*=118]. This mask was then used as a seed region in a psycho-physiological interaction (PPI) [7]. However, neither connectivity with any other hippocampal cluster [lowest FWE-corrected *p* of .353 at (-30, -21, -24); *t*-tests; *N*=118] nor a region in the amygdala [lowest FWE-corrected *p* of .435 at (-33, -6, -30) ; *t*-tests; *N*=118] or in the prefrontal cortex [lowest FWE-corrected *p* of .999 at (-39, 18, -6) ; *t*-tests; *N*=118] showed a significant relationship with changes in salivary E2.

For completeness sake, we also conducted a PPI analysis using the cluster showing a significant quadratic relationship to salivary E2 increase [(-15, -33, -12) thresholded at $p_{unc}<.001$; *t*-test; *N*=118] as the seed region. Neither connectivity with any other hippocampal cluster [lowest FWE-corrected *p* of .571 at (15, -33, -6); *t*-tests; *N*=118] nor an amygdala cluster [lowest FWE-corrected *p* of .953 at (36, -6, -21); *t*-tests; *N*=118] or a prefrontal cluster [lowest FWE-corrected *p* of .806 at (21, 24, 36); *t*-tests; *N*=118] showed a significant relationship with changes in salivary E2.

Supplementary Methods

Timeline of the study

The saliva samples (3 per day, 20-30 minutes apart) were drawn on Day 1 on average between ~6 pm and ~7 pm (before initial drug administration), on Day 2 between ~4.30 pm and ~6 pm and on Day 3 between ~6 pm and ~7.30 pm. Blood was drawn on average at ~6 pm on Day 1 and Day 2. Drug intake on Day 2 was on average around 7 pm and on Day 1 around 10.30 am. Scanning took place on average around 6 pm on Day 2.

Sample size calculation

Expecting a small to medium effect size of Cohen's f^2 = .10 at a statistical power of .80, a minimum N of 99 is required to detect an effect at a threshold of $p<.05$ in multiple regression analyses with two predictors (e.g. a linear and a quadratic increase in salivary E2). A slightly higher *N* of 125 was chosen in order to make sure that the sample size would be still adequate in the case of missing data points.

Recognition test instructions

Participants received the following written instructions: 'Now we will present the pictures that were presented yesterday ('old pictures') randomly mixed with pictures you haven't seen before ('new pictures'). For each picture, you will be asked to judge whether the picture is 'old' or 'new'. For this decision, you will have to choose between 6 options by selecting the corresponding box: 'absolutely sure old', 'relatively sure old', 'rather old', 'rather new', 'relatively sure new', 'absolutely sure new'. These options will be presented under the picture for every

decision. Please respond as accurately and quickly as possible. However, there is no time limit. Please try to use the whole scale. For each picture judged as 'old', you will be then asked whether you '**remember' details from the learning situation** or whether you just '**know' that the picture was presented yesterday**. Please choose **‘remember’** if you remember any detail from the learning situation such as what you have thought or felt when viewing the picture, at what time the picture was presented (before or after another specific picture) and so on. Please choose **‘know’** if you only remember that we presented the picture yesterday but not any further details from the learning situation.’

Arterial Spin Labeling (ASL)

The assessment of neural activity by fMRI relies on the blood-oxygen-level dependent (BOLD) effect, which is a temporal increase in oxyhemoglobin in active brain regions. This functional hyperemia is caused by an increase in blood flow to supply the tissue with glucose for aerobic glycolysis which is accompanied by a parallel decrease in the oxygen extraction fraction resulting in the increase in regional oxyhemoglobin levels. Because E2 has vasomodulatory effects and can dilate cerebral arteries[8] it could be possible that these unspecific, i.e. not task evoked effects, might influence the BOLD-effect. In particular, a greater baseline perfusion could result in a smaller relative task related increase, and could potentially bias thus the measure of neural activity associated to the memory task.

ASL was performed with the second version of quantitative imaging of perfusion using a single subtraction combined with thin-slice periodic saturation (Q2TIPS) as described in the work of Luh and colleagues (1999) [9], an echo-planar image readout, and proximal inversion with a control for off-resonance effects (PICORE) [10]. Because the hippocampus was our main region of interest, 14 contiguous axial slices were acquired covering mainly the medial temporal lobe (4 mm thickness with 1 mm gap; TI_1 0.7 s; TI_2, 1.1 s; thickness of inversion block 100 mm,

minimum gap between inversion block and slices 20 mm, thickness of stop saturation pulses 20 mm, TR 2.33 s; TE 11 ms; flip angle 90°; field of view 224 x 256; matrix 56 x 64, parallel acquisition acceleration factor 2, partial Fourier factor 7/8). 50 label and 50 control images were acquired yielding a total duration of the ASL measurement of 4 min 4 s including reference scans for parallel imaging. Relative CBF maps were calculated assuming an inversion efficiency of 95%, a blood T1 relaxation time of 1500 ms, and a blood/tissue water partition coefficient of 0.9 ml g^{-1}. ASL data of six participants were missing because of technical failure and excessive head movement (see Supplementary Table 12 for group affiliations).

Structural T1 maps were coregistered to functional images and segmented as described in the methods section of the main manuscript. Resulting tissue-class images were used to create individual flow fields employing the 'diffeomorphic anatomic registration through an exponentiated lie algebra algorithm' (DARTEL) algorithm. Flow fields were then used to normalize rCBF maps to Montreal Neurological Institute (MNI) space. Normalized rCBF maps were smoothed with a full-width half maximum Gaussian kernel of 8 mm for all directions. Linear and inverted u-shaped relationships were examined as described in the methods section of the main manuscript.

Supplementary References

1. Luisi, M. *et al.* Radioimmunoassay for progesterone in human saliva during the menstrual cycle. *Journal of Steroid Biochemistry* **14,** 1069–1073 (1981).
2. Fleming, S. M. & Lau, H. C. How to measure metacognition. *Front Hum Neurosci* **8,** 443 (2014).
3. Pruis, T. A., Neiss, M. B., Leigland, L. A. & Janowsky, J. S. Estrogen modifies arousal but not memory for emotional events in older women. *Neurobiology of Aging* **30,** 1296–1304 (2009).
4. Maki, P. M. & Resnick, S. M. Longitudinal effects of estrogen replacement therapy on PET cerebral blood flow and cognition. *Neurobiol. Aging* **21,** 373–383 (2000).
5. Ottowitz, W. *et al.* Evaluation of prefrontal–hippocampal effective connectivity following 24 hours of estrogen infusion: An FDG-PET study. *Psychoneuroendocrinology* **33,** 1419–1425 (2008).
6. Shaywitz, S. E. *et al.* Effect of Estrogen on Brain Activation Patterns in Postmenopausal Women During Working Memory Tasks. *JAMA* **281,** 1197–1202 (1999).
7. Friston, K. J. *et al.* Psychophysiological and modulatory interactions in neuroimaging. *Neuroimage* **6,** 218–229 (1997).
8. Duckles, S. P. & Krause, D. N. Cerebrovascular effects of oestrogen: multiplicity of action. *Clin. Exp. Pharmacol. Physiol.* **34,** 801–808 (2007).
9. Luh, W. M., Wong, E. C., Bandettini, P. A. & Hyde, J. S. QUIPSS II with thin-slice TI1 periodic saturation: a method for improving accuracy of quantitative perfusion imaging using pulsed arterial spin labeling. *Magn Reson Med* **41,** 1246–1254 (1999).
10. Wong, E. C., Buxton, R. B. & Frank, L. R. Implementation of quantitative perfusion imaging techniques for functional brain mapping using pulsed arterial spin labeling. *NMR Biomed* **10,** 237–249 (1997).
